# Sex Differences in the Trajectories of Cognitive Decline and Affected Cognitive Domains Among Older Adults With Controlled and Uncontrolled Glycemia

**DOI:** 10.1093/gerona/glae136

**Published:** 2024-05-22

**Authors:** Natália Cochar-Soares, Dayane C de Oliveira, Mariane M Luiz, Márlon J R Aliberti, Claudia K Suemoto, Andrew Steptoe, Cesar de Oliveira, Tiago S Alexandre

**Affiliations:** Department of Gerontology, Federal University of São Carlos, São Carlos, Brazil; Department of Physical Therapy, Federal University of São Carlos, São Carlos, Brazil; Department of Physical Therapy, Federal University of São Carlos, São Carlos, Brazil; Laboratory of Medical Research in Aging (LIM-66), Servico de Geriatria, Hospital das Clinicas, University of São Paulo Medical School, São Paulo, Brazil; Division of Geriatrics, Department of Internal Medicine, University of São Paulo Medical School, São Paulo, Brazil; Department of Behavioral Science and Health, University College London, London, UK; Department of Epidemiology and Public Health, University College London, London, UK; Department of Epidemiology and Public Health, University College London, London, UK; Department of Gerontology, Federal University of São Carlos, São Carlos, Brazil; Department of Epidemiology and Public Health, University College London, London, UK; (Medical Sciences Section)

**Keywords:** Aging, Cognition, Diabetes, Glycated hemoglobin, Longitudinal study

## Abstract

**Background:**

We aimed to analyze the trajectories of cognitive decline as a function of the presence of type 2 diabetes and glycemic control in analyzes stratified by sex in an 8-year follow-up period.

**Methods:**

A total of 1 752 men and 2 232 women aged ≥50 years who participated in the English Longitudinal Study of Ageing (ELSA), conducted from 2004 to 2012, were analyzed. The outcomes of interest were performance on the cognitive domains of memory, executive function, and temporal orientation as well as the global cognition score. Cognitive performance was standardized in *z*-scores in strata based on schooling and age. The participants were classified as without diabetes, with controlled glycemia, and with uncontrolled glycemia, according to medical diagnosis, glucose-lowering medications use and HbA1c levels. Generalized linear mixed models controlled by sociodemographic, behavioral, and health-related characteristics were used for the trajectory analyses.

**Results:**

No differences in *z*-scores were found for global cognition or cognitive domains based on diabetes classification in men and women at baseline. More than 8 years of follow up, women with uncontrolled glycemia had a greater decline in *z*-scores for global cognition (−0.037 *SD*/year [95% CI: −0.073; −0.001]) and executive function (−0.049 *SD*/year [95% CI: −0.092; −0.007]) compared with those without diabetes. No significant difference in trajectories of global cognition or any cognitive domain was found in men as a function of diabetes classification.

**Conclusions:**

Women with uncontrolled glycemia are at greater risk of a decline in global cognition and executive function than those without diabetes.

Cognitive decline can occur with aging as a result of changes to the central nervous system, involving a reduction in the volume of the cerebral, hippocampal, prefrontal, and temporal cortices, which are important areas for the adequate functioning of cognitive domains, such as memory, executive function, temporal orientation and global cognition (1).

The occurrence of chronic diseases, such as type 2 diabetes mellitus, can aggravate brain damage and cognitive decline. For example, chronic hyperglycemia due to diabetes increases the presence of inflammatory cytokines in the brain, causing vascular lesions that contribute to plaque formation (atheroma) in blood vessels and possibly compromised brain circulation. Also, hypoglycemia, a common event in those with uncontrolled glycemia, can cause neuronal death (2).

Although some studies have analyzed the association between diabetes and cognitive function and its decline over time, conflicting results are reported regarding the most affected cognitive domains. For example, Zheng and collaborators (2018) found a decline in *z*-scores of global cognition, memory, and executive function with each 1-mmol/mol increase in HbA1c (3), whereas Callisaya et al. found an association between diabetes and declines in memory and verbal fluency (4).

With regards to glycemic control, Zhang et al. found that individuals with uncontrolled glycemia performed worse in the executive function domain compared with those with controlled glycemia (5). A review study demonstrated that type 2 diabetes is associated with poor performance on information processing speed, memory, attention, executive function, and global cognition in longitudinal studies, but emphasized that few studies have analyzed this association for a period longer than 6 years, and most did not consider the control of the disease (6).

A meta-analysis involving 15 cross-sectional and longitudinal studies showed that individuals with diabetes had poorer performances in the cognitive domains of memory and executive function (7). However, many of the studies reviewed only used self-reported type 2 diabetes, did not consider the glycemic control of the disease, cognitive performance was not standardized by *z*-scores for age and education and the analyzes were not controlled for important variables, such as lifestyle and clinical conditions (heart disease, stroke, cholesterol levels, and obesity).

In addition to these gaps in knowledge, there is no consensus in the literature on differences between the sexes regarding the association of type 2 diabetes on the performance of cognitive function. Some studies have shown that men have a more accentuated decline in cognitive function compared with women due to possible neuroprotection from female hormones (8,9). However, other studies report that women are at greater risk due to the greater likelihood of developing macrovascular complications and the greater difficulty in maintaining glycemia levels within the standards of normality due to the higher prevalence of obesity (10,11).

Therefore, the present study aimed to test the following hypotheses: (1) only uncontrolled glycemia increases the risk of cognitive decline and (2) differences exist between the sexes in the association between uncontrolled glycemia and the decline in global cognitive function and specific cognitive domains, with women being more affected than men.

## Method

### Study Population

The data used in the present study are from the English Longitudinal Study of Ageing (ELSA), an ongoing panel study involving community-dwelling older English adults ≥50 years old. ELSA began in 2002 with a nationally representative cohort selected by stratified random probabilistic sampling (12). Follow-up interviews occur every 2 years and health examinations every 4 years. The first health examination occurred in 2004–2005. A detailed description of the study can be found in a previous publication (13).

**Figure 1.**
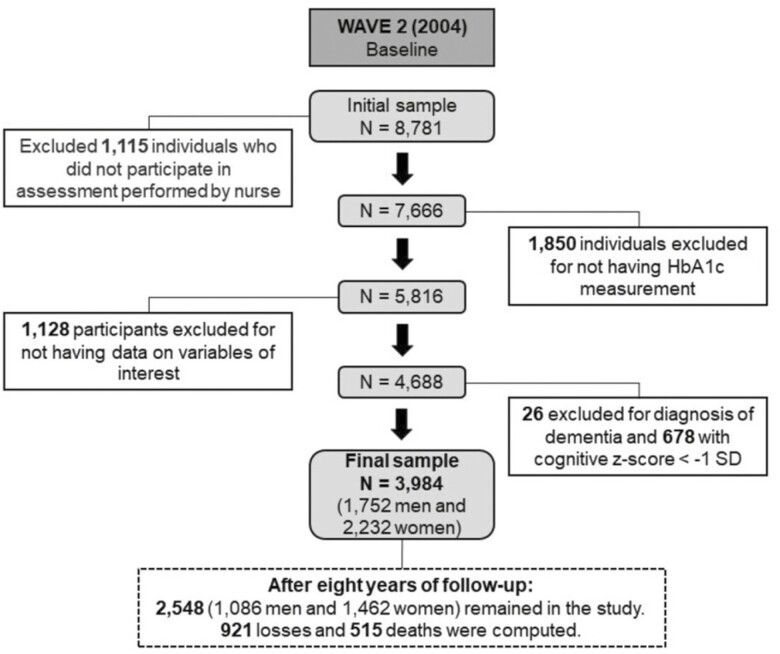
Study sample flowchart.

Figure 1 displays the sample flowchart for the present study, for which the 2004–2005 assessment was considered the baseline. A total of 3 984 participants ≥50 years old were included (1 752 men and 2 232 women) and they were reevaluated after 4 and 8 years.

### Cognitive Function

The cognitive domains of memory, executive function, and temporal orientation, as well as global cognition, were assessed. Cognitive measures used by ELSA in this study were developed by the U.S. Health and Retirement Study (14).

Memory was assessed using the word list test, on which the participants heard a list of 10 words, which they were immediately asked to recall. After approximately 2 min, the participants were asked to recall as many of the same 10 words as possible. The score was the sum of correctly recalled words (1 point per word) in the 2 attempts, and so the total score ranged from 0 to 20 points (15).

Executive function was assessed using the verbal fluency test. The participants were asked to name the maximum number possible of different animals they could remember in 1 min. The total was the sum of the number of animals cited, with higher numbers indicating a better performance (16,17).

Temporal orientation was assessed using a questionnaire with 4 questions about month, year, week day and month day, with 1 point awarded for each correct answer (range: 0–4 points) (17).

The sum of the points on the 3 tests, which evaluated each of the cognitive domains (memory, executive function, and temporal orientation), constituted the global cognition score with higher scores denoting better cognitive function.

The dependent variables (memory, executive function, temporal oriental, and global cognition) were expressed as standardized *z*-scores, which are widely used in longitudinal studies that evaluate cognitive function (3,4). z-Scores were calculated for each age stratum (50–59, 60–69, 70–79, and 80 years or older) and education stratum (English classification: 0–11, 12–13, and 14 years or more), as the strong influence of these variables on cognitive performance is well established (18,19). To enable comparisons and determine decline over time, mean performance, and standard deviation (SD) values from baseline for each age and education strata were considered in the calculation of the other waves of the ELSA Study.

### Diabetes Mellitus

Participants who reported not having diabetes did not take medication for diabetes and had HbA1c levels <6.5% were classified as “without diabetes.” Participants were classified as “with controlled glycemia” when reported having diabetes, took or did not take medication for diabetes and had HbA1c <7%, or if they did not report having diabetes, did not take medication for diabetes, but had HbA1c between 6.5% and 7%, or if they did not report having diabetes, but took medication for diabetes and had HbA1c <7%. Individuals were classified as “with uncontrolled glycemia” when reported having diabetes, took or did not take medication for diabetes and had HbA1c ≥7%, or if they did not report having diabetes, took or did not take medication for diabetes, but had HbA1c ≥7% (20).

### Covariates

Control variables were those associated with diabetes and/or cognitive decline reported in the literature (20). The sociodemographic covariates were age (in years, centralized and fixed on the baseline) and household wealth (financial wealth, housing wealth, and physical wealth [jewelry and works of art]) divided into quintiles.

The behavioral variables were physical activity, smoking, and alcohol intake. Physical activity level was determined using 3 questions about the frequency and intensity of physical activity taken from the Physical Activity and Sedentary Behavior Assessment Questionnaire, validated by the Health Survey for England (21). The participants were classified as active (moderate or vigorous physical activity more than once per week) or inactive (moderate or vigorous physical activity once per week or less or any frequency of light activity) (22). In terms of smoking, participants were classified as nonsmokers, exsmokers, or smokers. For alcohol intake, participants were classified as nondrinkers, until once per week, 2–6 times per week, or daily (23).

Health status included self-reported diagnoses of heart disease, stroke, and hypertension, because are the main complications triggered by diabetes (24–29).

Waist circumference was measured twice using a flexible metric tape at the midpoint between the lowest rib and upper edge of the iliac crest. The average of the 2 measures was used. Abdominal obesity was recorded when the waist circumference was >102 cm for men and >88 cm for women (30).

Triglyceride levels were considered high when ≥150 mg/dl, low-density lipoprotein (LDL) was considered high when ≥100 mg/dl and high-density lipoprotein (HDL) was considered low when <40 mg/dl for men and <50 mg/dl for women (31).

### Statistical Analysis

Descriptive analysis was performed for the sample characterization. Differences in baseline characteristics according to the presence of diabetes and glycemic control were analyzed using the chi-squared test, analysis of variance (ANOVA), and Tukey’s post hoc test. Differences in the characteristics of the interviewees more than 8 years of follow-up, those who had died and those lost to follow up were analyzed using chi-squared and *t*-test. A *p*-value *p* < .05 was considered statistically significant. Generalized linear mixed models were used to estimate the trajectories of global cognition and specific domains over time. For such, the XTMIXED in Stata 14 SE (StataCorp LLC, College Station, TX) was employed. Generalized linear mixed models are ideal for studies that work with repeated measures, enabling the statistical modeling of time-dependent changes in the outcome and the strength of associations between variables (32).

Since sex differences have been reported for diabetes and cognitive function in literature (8–11,33,34), an interaction analysis between sex and diabetes concerning cognitive performance was performed. The interaction between female sex and uncontrolled glycemia was significant for a greater decline in *z*-scores of global cognition (−0.075 *SD*/year [95% CI: −0.123; −0.027]) and executive function (−0.081 *SD*/year [95% CI: −0.133; −0.028]). Therefore, the analyzes were stratified by sex to identify possible differences in cognitive function trajectories between men and women.

Decline rates of cognitive domains and global cognition were compared using ß coefficients and 95% confidence intervals (CI). In the final model, the intercept represents the estimated mean difference in global cognitive performance or performance in a specific cognitive domain analyzed in *z*-scores at baseline among the participants according to the presence of diabetes and glycemic control, taking individuals without diabetes as the reference category. On the slope, time (in years) indicates the magnitude of the trajectory of decline in global cognition or in a specific cognitive domain analyzed independently of the covariates (as if time per se were the determinant of decline). The interaction between time and the diabetes classification represents the estimated difference in the annual rate of decline in global cognition or a specific cognitive domain (slope) in each of the 2 groups (controlled glycemia and uncontrolled glycemia) compared with the reference group (without diabetes).

Inverse probability weighting was used to correct for survival bias and attenuate the impact of losses to follow-up, common in longitudinal studies. This method calculates the probability of participation and survival of the individuals during the study follow up, incorporating this probability into the analyzes (35).

### Ethical Aspects

All participants signed a statement of informed consent. Ethical approval for all waves of the ELSA was granted by the London Multicentre Research and Ethics Committee (MREC 01/2/91).

## Results

Among the 3 984 participants at baseline (1 752 men and 2 232 women), 3 192 (1 380 men and 1 812 women), and 2 548 (1 086 men and 1 462 women) were reevaluated after 4 and 8 years, respectively. Therefore, 64% of the initial analytical sample participated in the 3 waves of the study and 80% participated in 2 waves. At baseline, the mean age of men was 65.4(±9.0) years for the group without diabetes, 68.2(±8.7) for the group with controlled glycemia, and 66.9(±8.5) for those with uncontrolled glycemia, and the prevalence of diabetes was 10.1%. Among women, the mean age was 65.6(±9.1) years for the group without diabetes, 68.7(±9.2) for those with controlled glycemia, and 67.5(±9.2) for uncontrolled glycemia, and the prevalence of diabetes was 6.4%. The baseline characteristics according to diabetes classification are displayed in Tables 1 and 2.

**Table 1.** Sociodemographic and Behavioral Characteristics of 1 752 men and 2 232 Women who Participated in ELSA Study at Baseline According to Diabetes Classification

	Men			Women		
Sociodemographic	Without diabetes (*n* = 1 574)	Controlled glycemia (HbA1c < 7%) (*n* = 103)	Uncontrolled glycemia (HbA1c ≥ 7.0%) (n = 75)	Without diabetes (*n* = 2 088)	Controlled glycemia (HbA1c < 7%) (*n* = 92)	Uncontrolled glycemia (HbA1c ≥ 7.0%) (*n* = 52)
Age, years (*SD*)	65.4 (9.0)	**68.2 (8.7)** ^a^	66.9 (8.5)	65.6 (9.1)	**68.7 (9.2)** ^a^	67.5 (9.2)
Schooling, (%)						
14 years or more	33.7	32.1	36.0	22.1	14.1	13.5
12–3 years	24.6	29.1	16.0	25.5	22.8	17.3
0–11 years	41.7	38.8	48.0	52.4	63.1	**69.2** ^a^
Household wealth (quintiles), (%)						
Highest quintile	26.5	20.4	21.3	25.1	16.3	**9.6** ^a^
2nd quintile	25.3	20.4	21.3	21.7	14.1	17.3
3rd quintile	21.0	17.5	17.4	21.1	22.8	19.2
4th quintile	16.1	22.3	21.3	18.3	22.9	28.9
Lowest	11.1	19.4	18.7	13.8	23.9	25.0
Behavioral						
Alcohol intake, (%)						
Rarely or never	8.6	14.6	**21.3** ^a^	19.2	**38.0** ^a^	25.0
Up to once per week	12.7	19.4	21.3	20.3	18.5	26.9
2–6 times per week	47.7	41.7	38.7	41.1	**28.3** ^a^	**25.0** ^a^
Daily	23.5	16.5	**12.0** ^a^	13.3	**6.5** ^a^	9.6
Did not answer	7.5	7.8	6.7	6.1	8.7	13.5
Smoking, (%)						
Nonsmoker	30.1	22.3	21.3	46.7	39.1	50.0
Ex-smoker	59.2	68.0	65.3	43.0	51.1	36.5
Smoker	10.7	9.7	13.4	10.3	9.8	13.5
Physical activity (inactive), (%)	26.7	**44.6** ^a^	38.7	31.7	**51.1** ^a^	42.3

*Notes*: Data expressed as percentage, mean, and standard deviation (SD); Statistical significance *p* < .05. ELSA = English Longitudinal Study of Ageing.

^a^Significantly different from individuals without diabetes.

^b^Significantly different from individuals with controlled glycemia.

**Table 2.** Clinical, Anthropometric, and Biochemical Characteristics of 1 752 men and 2 232 Women who Participated in ELSA Study at Baseline According to Diabetes Classification

	Men			Women		
Health conditions	Without diabetes (*n* = 1 574)	Controlled glycemia (HbA1c < 7%) (*n* = 103)	Uncontrolled glycemia (HbA1c ≥ 7.0%) (*n* = 75)	Without diabetes (*n* = 2 088)	Controlled glycemia (HbA1c < 7%) (*n* = 92)	Uncontrolled glycemia (HbA1c ≥ 7.0%) (*n* = 52)
Cardiovascular disease (yes), (%)	19.8	**32.0** ^a^	29.3	17.2	**29.3** ^a^	26.9
Stroke (yes), (%)	3.1	**9.7** ^a^	2.7	2.4	8.7	7.7
Hypertension (yes), (%)	36.9	**68.9** ^a^	**56.0** ^a^	38.5	**72.8** ^a^	**71.1** ^a^
Cognitive performance, (*SD*)						
Temporal orientation *z*-score	−0.03 (1.0)	0.08 (0.9)	−0.05 (1.0)	0.14 (0.8)	0.11 (0.8)	0.12 (0.7)
Executive function *z*-score	0.27 (0.8)	0.28 (1.1)	0.37 (0.8)	0.19 (0.8)	0.14 (1.1)	0.32 (1.0)
Memory *z*-score	0.44 (0.8)	0.11 (0.8)	**−0.17 (0.9)** ^a^	0.33 (0.9)	0.24 (0.9)	0.12 (0.8)
Global cognition *z*-score	0.25 (0.8)	0.30 (1.0)	0.25 (0.7)	0.31 (0.8)	0.26 (1.0)	0.33 (0.9)
Anthropometric						
Waist circumference, (*SD*)	100.1 (10.6)	**105.6 (13.0)** ^a^	**107.8 (12.2)** ^a^	89.2 (11.5)	**101.3 (13.2)** ^a^	**97.9 (13.8)** ^a^
>102 cm men >88 cm women, (%)	40.5	**56.3** ^a^	**64.0** ^a^	51.0	**90.2** ^a^	**73.1** ^a^
Biochemical						
HDL, (*SD*)	54.6 (12.8)	**47.2 (13.2)** ^a^	**46.7 (10.8)** ^a^	65.2 (14.3)	**55.2 (12.2)** ^a^	**53.8 (12.3)** ^a^
<40 mg/dL men <50 mg/dL women, (%)	10.7	**29.1** ^a^	**25.3** ^a^	10.1	**33.7** ^a^	**36.5** ^a^
LDL, (*SD*)	136.5 (36.7)	**103.9 (36.9)** ^a^	**100.8 (35.7)** ^a^	147.8 (37.4)	**117.9 (36.4)** ^a^	**104.1 (32.9)** ^a.b^
≥100 mg/dl, (%)	85.1	**50.5** ^a^	**44.0** ^a^	91.2	**66.3** ^a^	**40.4** ^a.b^
Triglycerides, (SD)	151.0 (74.5)	**170.4 (77.6)** ^a^	**175.1 (72.6)** ^a^	139.3 (65.4)	**175.2 (71.4)** ^a^	**181.6 (74.0)** ^a^
≥150 mg/dl, (%)	43.4	55.3	**62.7** ^a^	35.7	**60.9** ^a^	**63.**5^a^
HbA1c, (*SD*)	5.4 (0.3)	**6.2 (0.5)** ^a^	**8.2 (1.3)** ^a.b^	5.4 (0.3)	**6.3 (0.5)** ^a^	**8.2 (1.2)** ^a.b^

*Notes*: Data expressed as percentage, mean, and standard deviation (SD); Statistical significance *p* < .05. ELSA = English Longitudinal Study of Ageing.

^a^Significantly different from individuals without diabetes.

^b^Significantly different from individuals with controlled glycemia.

Men with uncontrolled glycemia consumed less alcohol and had a lower memory *z*-score compared with men without diabetes. Men with controlled glycemia were older, less physically active, and had more cardiovascular disease than those without diabetes. Men with controlled glycemia and uncontrolled glycemia had more hypertension, abdominal obesity and triglyceride levels, and lower HDL and LDL levels than those without diabetes (Tables 1 and 2).

Women with uncontrolled glycemia had less education and lower household wealth compared with those without diabetes. Women with controlled glycemia were older, less physically active, and had more cardiovascular diseases compared with those without diabetes. Women with diabetes (controlled or not) consumed less alcohol, had more hypertension, more abdominal obesity and triglycerides, and lower levels of HDL and LDL compared with those without diabetes. The only different characteristic between women with uncontrolled glycemia and with controlled glycemia was the lower levels of LDL among those with uncontrolled glycemia (Tables 1 and 2).

The individuals who had died and those lost to follow up were mainly male, older, had less income and schooling, smoked less, consumed less alcohol, were more physically inactive, had a higher prevalence of heart disease, stroke, and hypertension, lower *z*-score of executive function, memory and global cognition, as well as had higher waist circumference, lower HDL and LDL levels and a higher level of HbA1c when compared with the individuals who remained until the end of the follow up (Supplementary Table 1).

Table 3 shows the estimates of the generalized linear mixed models for the intercept (baseline) and changes in cognitive function according to diabetes classification per year more than the 8-year follow-up period. For men, no differences in cognitive function were found according to diabetes classification at baseline. Additionally, no differences were found regarding the rates of decline in cognitive *z*-scores among men according to diabetes classification. For women, no differences in cognitive function were found according to diabetes classification at baseline. Also, no differences were found regarding the rates of decline in cognitive z-scores between the groups with controlled glycemia and without diabetes. In contrast, women with uncontrolled glycemia had a greater annual decline rate more than the 8-year follow up for global cognition (−0.037 *SD*/year; 95% CI: −0.073 to −0.001; *p* < .05) and executive function z-scores (−0.049 *SD*/year; 95% CI: −0.092 to −0.007; *p* < .05) than women without diabetes. No differences between these groups were found regarding the decline rates of temporal orientation and memory domains (Table 3).

**Table 3.** Adjusted Generalized Linear Mixed Models for Trajectory of Cognitive Performance in 8-year follow up According to Diabetes Classification in 1 752 men and 2 232 Women who Participated in ELSA Study (2004–2012)

	Men	Women
	*n* = 1 752	*n* = 2 232
Model 1—Global cognition	Estimated parameters (95% CI)	
Intercept		
Without diabetes	Reference	Reference
Controlled glycemia	0.016 (−0.144; 0.177)	−0.094 (−0.299; 0.110)
Uncontrolled glycemia	0.049 (−0.106; 0.204)	0.118 (−0.113; 0.350)
Slope		
Time, years	−0.105 (−0.196; −0.013)*	−0.023 (−0.102; 0.055)
Time × without diabetes	Reference	Reference
Time × controlled glycemia	−0.001 (−0.029; 0.028)	0.012 (−0.019; 0.044)
Time × uncontrolled glycemia	0.032 (−0.001; 0.065)	−0.037 (−0.073; −0.001)*
Model 2—Memory		
Intercept		
Without diabetes	Reference	Reference
Controlled glycemia	−0.071 (−0.228; 0.084)	−0.200 (−0.380; −0.021)
Uncontrolled glycemia	−0.128 (−0.322; 0.064)	−0.031 (−0.254; 0.192)
Slope		
Time, years	−0.027 (−0.101; 0.046)	−0.161 (−0.297; −0.025)*
Time × without diabetes	Reference	Reference
Time × controlled glycemia	0.019 (−0.010; 0.050)	0.028 (−0.005; 0.062)
Time × uncontrolled glycemia	0.015 (−0.028; 0.059)	0.003 (−0.042; 0.049)
Model 3—Temporal orientation		
Intercept		
Without diabetes	Reference	Reference
Controlled glycemia	0.048 (−0.128; 0.225)	−0.058 (−0.217; 0.100)
Uncontrolled glycemia	−0.060 (−0.286; 0.164)	−0.133 (−0.342; 0.074)
Slope		
Time, years	−0.081 (−0.274; 0.111)	−0.023 (−0.066; 0.019)
Time × without diabetes	Reference	Reference
Time × controlled glycemia	0.001 (−0.037; 0.037)	0.013 (−0.020; 0.048)
Time × uncontrolled glycemia	0.003 (−0.046; 0.053)	0.008 (−0.035; 0.052)
Model 4—Executive function		
Intercept		
Without diabetes	Reference	Reference
Controlled glycemia	0.011 (−0.158; 0.180)	−0.019 (−0.251; 0.213)
Uncontrolled glycemia	0.108 (−0.060; 0.278)	0.169 (−0.105; 0.444)
Slope		
Time, years	−0.104 (−0.203; −0.005)*	0.073 (0.030; 0.116)*
Time × without diabetes	Reference	Reference
Time × controlled glycemia	−0.005 (−0.035; 0.025)	0.007 (−0.027; 0.042)
Time × uncontrolled glycemia	0.031 (−0.001; 0.062)	−0.049 (−0.092; −0.007)*

*Notes*: Cognitive performance on each test is standardized by *z*-score. Global cognition is calculated by average *z*-scores of cognitive tests and subsequently standardized by mean. Models adjusted by sociodemographic characteristics (age and household wealth), behavioral habits (alcohol intake, smoking, and physical activity), health conditions (cardiovascular disease, stroke, and hypertension), anthropometric measures (waist circumference), and biochemical measures (HDL cholesterol, LDL cholesterol, and triglycerides). ELSA = English Longitudinal Study of Ageing.

*p < .05.

According to the estimated coefficients, the estimated change over time was stable, in other words, there was no significant decline in memory and temporal orientation *z*-scores in men and global cognition and temporal orientation in women for a 1-unit increase in time for the reference group (when all other covariates in the model were zero at average values): aged 50 years at baseline, household wealth = 5th quintile, those who remained without diabetes, nondrinkers, nonsmokers, nonsedentary lifestyle, no hypertension, no cardiovascular disease, no stroke, no abdominal obese and with normal levels of HDL, LDL, triglycerides, and HbA1c. For the same group mentioned earlier, there was a decline over time in global cognition and executive function *z*-scores in men and memory *z*-scores in women, as well as an increase in executive function *z*-scores in women (Table 3).

**Figure 2.**
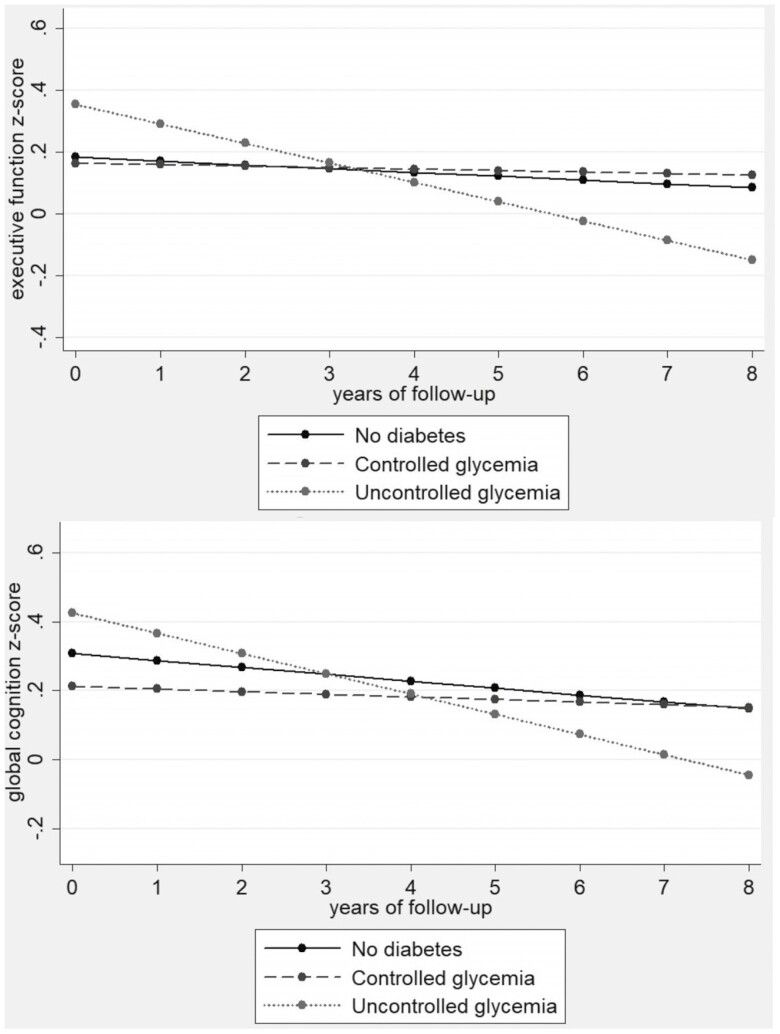
Trajectory graphs of global cognition and executive function in women throughout 8-year follow up according to diabetes classification (*n* = 2 232), ELSA Study, 2004–2012.

Figure 2 displays the mean predicted *z*-scores for performance on global cognition and executive function for the groups without diabetes, with controlled glycemia, and with uncontrolled glycemia each year during the 8-year follow up of the 2 232 English women participants.

## Discussion

The main finding of the present study showed that only women with uncontrolled glycemia presented greater declines in global cognition and executive function compared with women without diabetes. The decline in executive function may have contributed to the decline in global cognition, due to the global cognition score to be constituted by the sum of points on the 3 tests, which assessed the cognitive domains.

In contrast, no differences in the trajectory of global and specific cognitive *z*-scores were found over time between women with controlled glycemia and those without diabetes. Furthermore, we did not observe differences in the trajectory of global and specific cognitive *z*-scores for men as a function of diabetes classification. Therefore, our study not only highlights the importance of controlling diabetes but also reveals the greater vulnerability of women to cognitive decline when glycemia is not controlled.

Investigating sex differences in cognitive decline, Levine and collaborators (2021) assessed 26 088 individuals followed up for nearly 8 years. Although not studying exposure to diabetes, the authors found that women had a significantly faster decline in global cognition and executive function compared with men. In contrast, the authors found no difference in memory decline between the sexes (33). The greater likelihood of cognitive changes in women seems to be mediated by the reduction in estrogen levels beginning with menopause, the greater likelihood of women having small vessel disease, the fact that women have a lower volume of gray matter compared with men and the fact that women have a longer life expectancy, but with multimorbidity and disabilities (33,36). This set of factors may generate a greater predisposition in women to the cerebral atrophy that occurs with the aging process and an increased risk of developing neurodegenerative diseases with the advance in age (33).

Besides hormonal and biological factors, other gender-associated factors—mediated by social and behavioral aspects throughout life—can explain different trajectories of cognition in women and men as they age. For instance, older men had better access to higher education and intellectual job positions and participated more in sports activities than women. Together, such aspects may ensure greater cognitive reserve in men than in women (37). The increased brain vulnerability of women could be accentuated in the presence of uncontrolled glycemia, increasing their risk for cognitive decline (11,38).

Palarino, Boardman, and Rogers (2023) examined sex differences in the association between diabetes and cognitive function using longitudinal data with 20 years of follow up of 19 190 women and 15 580 men residing in the United States. The authors found that women had a more accentuated decline in cognitive function compared with men and this decline was exacerbated by diabetes (39). However, cognitive domains were not assessed separately, and the occurrence of diabetes was based only on self-reports, which hinders comparisons to the present results.

In a longitudinal study with an 8-year follow-up period using data from the ELSA Study, Zaninotto et al. explored the factors that influence the cognitive decline and whether these differed by gender and found that women had significantly less decline than men in memory (0.011, *SE* 0.006), executive function (0.012, *SE* 0.006) and global cognitive function (0.016, *SE* 0.004) (40). However, the authors did not consider glycemic control, as performed in the present investigation. These methodological divergences may explain the different results with regards to memory. Another possible explanation for not having found an association between uncontrolled glycemia and memory decline in women in the present study resides in the hypothesis that the decline in global cognition and executive function precedes memory decline, as found in population-based studies involving older women (41).

Additionally, type 2 diabetes is associated with cardiovascular risk factors, increased incidence of cerebrovascular disease, and non-amnesic forms of cognitive impairment, such as vascular dementia. Therefore, it was not surprising that individuals with uncontrolled glycemia had a greater decline in executive function and language skills, but not in memory (42,43).

Physiopathologically, the greater decline in global cognition and executive function in women with uncontrolled glycemia compared with those with controlled glycemia or without diabetes is related to the presence of inflammatory factors resulting from chronic hyperglycemia, which can lead to changes in structures related to cognitive functioning, such as the hippocampus and prefrontal cortex (7). Cerebral atrophy caused by uncontrolled glycemia and the consequent reduction in neural and glial cells tend to be stabilized by glycemic control, as insulin plays an important role in maintaining cognitive performance. This pathway might explain why women with uncontrolled glycemia are more affected (44). Moreover, as type 2 diabetes affects the organism jointly with cardiometabolic disorders, the fact that women generally have a larger waist circumference and higher frequency of cardiovascular events may result in worse cognitive consequences in the female sex compared with the male sex when exposed to high glycemic levels (11,34,45).

As we did not find an association between controlled glycemia and cognitive decline in women and this association only occurred in uncontrolled glycemia, our results suggest that 7 per se may negatively affect cognitive function. Dove et al. (2021) followed up 2 522 individuals for 12 years and also found that uncontrolled glycemia (HbA1c ≥7.5%) was associated with a twofold greater risk of cognitive impairment, underscoring the importance of glycemic control in individuals with type 2 diabetes (45).

The strong points of this study include its large representative sample of the English population aged 50 and older, which enabled adjusting the analyzes by several control variables, the classification of individuals according to the presence of diabetes and glycemic control in addition to stratification by sex. Moreover, the use of generalized linear mixed models with data from 3 waves of the ELSA Study enabled the longitudinal evaluation of the trajectory of global and specific cognitive domains throughout a long follow-up period. Another strong point was the classification of diabetes based on glycated hemoglobin, which is considered a reliable measure, as it furnishes a 3-month weighted mean of glycemia. Lastly, the evaluation of cognitive domains besides the use of a global measure of cognition enabled identifying the domains most impacted by uncontrolled glycemia.

The limitations include losses to follow up, which is an unavoidable source of bias in longitudinal studies. The missing data from the second and third waves of the study could also be considered an important limitation, since those who died and those lost in the follow up were mainly male, with worse sociodemographic and health characteristics as well as with lower *z*-score of executive function, memory, and global cognition. However, the use of Inverse Probability Weighting in researchers analyzes, where a weight was assigned to each participant according to the calculation of their probability of participation and survival during the study follow up, can attenuate the survivorship and participation bias. Another limitation was the smaller number of participants with diabetes. Despite this limitation, it was possible to identify an association between uncontrolled glycemia and cognitive decline in women during the 8-year follow-up period. The noninclusion of the time elapsed since the diagnosis of diabetes can be considered another limitation of this study. Lastly, the ELSA study only involved community-dwelling individuals, which impedes estimates for institutionalized individuals, who tend to have a higher prevalence of diabetes and greater cognitive impairment.

## Conclusion

The present findings provide evidence that women who do not adequately control glycemia are at greater risk of a decline in global cognition and executive function compared with women without diabetes. Moreover, sex seems to influence this association, as no difference in the trajectory of cognitive function was found in men according to diabetes classification. Notably, individuals with controlled glycemia, independently of sex, did not present any decline in global cognition or any of the cognitive domains.

Therefore, glycemic control may contribute to the maintenance of executive function and global cognition, especially in women. These findings highlight the importance of the implementation of public health promotion programs involving early diagnosis, patient adherence to treatment, and the transmission of knowledge regarding diabetes to assist in preventing possible complications resulting from the disease, including cognitive decline.

## Supplementary Material

glae136_suppl_Supplementary_Table

## Data Availability

The data that support the findings of this study are available by request from the corresponding author. The data from the English Longitudinal Study of Ageing (ELSA), that underlie the results reported in this study, are available from the UK Data Service for researchers who meet the criteria for access to confidential data under conditions of the End User License: http://ukdataservice.ac.uk/media/455131/cd137-enduserlicence.pdf. The data can be accessed from https://www.elsa-project.ac.uk/accessing-elsa-data and can be requested through the website https://ukdataservice.ac.uk/help/.
