# Supplementary material for: Sex Differences in the Trajectories of Cognitive Decline and Affected Cognitive Domains Among Older Adults With Controlled and Uncontrolled Glycemia
Source: J Gerontol A Biol Sci Med Sci. 2024 May 22;79(7):glae136. doi: 10.1093/gerona/glae136 (PMC11181940; doi:10.1093/gerona/glae136)
Supplement: glae136_suppl_Supplementary_Table [file glae136_suppl_supplementary_table.docx]

**eTable 1 – Comparison of baseline characteristics between individuals lost to follow-up and those who remained in the study over eight-year follow-up (ELSA Study, 2004 – 2012).**

|  | **Participants who remained in the study (n = 2,548)** | **Participants who lost to follow-up**  **(n = 1,436)** |
| --- | --- | --- |
| Sex, female | 57.4***** | 53.6***** |
| Age, years (SD) | 64.1 (8.1)***** | 68.6 (10.0)***** |
| Household wealth (quintiles), (%) |  |  |
| Highest quintile | 27.8***** | 20.3***** |
| 2^nd^ quintile | 24.0 | 20.7 |
| 3^rd^ quintile | 20.5 | 21.6 |
| 4^th^ quintile | 16.4***** | 20.3***** |
| Lowest | 11.3* | 17.1***** |
| Schooling, (%) |  |  |
| 14 years or more | 31.0* | 19.7***** |
| 12 to 13 years | 27.3***** | 20.6***** |
| 0 to 11 years | 41.7* | 59.7***** |
| Smoking, (%) |  |  |
| Non-smoker | 41.2* | 34.7***** |
| Ex-smoker | 48.8* | 53.7* |
| Smoker | 10.0 | 11.6 |
| Alcohol intake, (%) |  |  |
| Rarely or never | 14.2***** | 17.8***** |
| Up to once per week | 18.2 | 15.7 |
| 2 to 6 times per week | 44.7 | 40.5 |
| Daily | 17.9 | 15.8 |
| Did not answer | 5.0* | 10.2* |
| Physical activity (inactive), (%) | 27.5***** | 36.7***** |
| Cardiovascular disease (yes), (%) | 16.9***** | 23.4***** |
| Stroke (yes), (%) | 2.4***** | 4.3***** |
| Hypertension (yes), (%) | 37.1* | 45.6* |
| Cognitive performance, (SD) |  |  |
| Temporal orientation z-score | 0.1 (0.9) | 0.1 (0.9) |
| Executive function z-score | 0.3 (0.9)* | 0.2 (0.8)* |
| Memory z-score | 0.2 (0.9)* | 0.1 (0.9)* |
| Global cognition z-score | 0.3 (0.9)* | 0.2 (0.8)* |
| Waist circumference, (SD) | 94.2 (12.6)* | 95.4 (13.0)* |
| >102 cm men >88 cm women, (%) | 47.7 | 49.8 |
| HDL, (SD) | 60.5 (15.0)***** | 58.6 (14.3)***** |
| <40 mg/dL men <50 mg/dL women, (%) | 11.7 | 12.6 |
| LDL, (SD) | 142.4 (38.4)***** | 136.0 (38.8)***** |
| ≥ 100 mg/dl, (%) | 87.5***** | 82.1***** |
| Triglycerides, (SD) | 145.5 (70.4) | 149.1 (70.9) |
| ≥ 150 mg/dl, (%) | 40.4 | 41.2 |
| HbA1c, (SD) | 5.5 (0.6)* | 5.6 (0.7)* |

Chi-squared test was performed for categorical variables; t test was performed for continuous variables to evaluate differences in baseline characteristics between individuals lost to follow-up and those who remained in the study. Data expressed as percentage, mean and standard deviation (SD). *Statistical significance: p-value <0.05.
